# MF-TLMLP: A multi-channel fusion-based multi-fidelity transfer learning method for predicting the cutting performance of conical picks

**DOI:** 10.1371/journal.pone.0359537

**Published:** 2026-10-23

**Authors:** Meng Zhou, Xiaofei Song, Lingtao Weng, Weiguo Gao, Ketong Dong, Guandong Lu, Ruilin Ni, Dawei Zhang

**Affiliations:** 1 Key Laboratory of Mechanism Theory and Equipment Design of Ministry of Education, Tianjin University, Tianjin, China; 2 Sany Heavy Equipment International Holdings Co., Ltd., Shenyang, China; Henan Polytechnic University, CHINA

## Abstract

Conical picks are critical components in key excavation equipment (including shearers, continuous miners, and roadheaders) employed for rock fragmentation in mining and civil engineering. This study proposes the multi-fidelity transfer learning multi-layer perceptron (MF-TLMLP) method to predict cutting performance. Addressing the scarcity of high-fidelity (HF) data, MF-TLMLP integrates low-fidelity (LF) and HF data through a multi-channel fusion and transfer learning approach. Simulations using Latin hypercube sampling (LHS) generated 2500 LF samples and 500 HF samples. Compared to traditional Multi-Layer Perceptron (MLP) and transfer learning models, MF-TLMLP demonstrated superior performance, achieving a higher coefficient of determination (R^2^) and a lower mean squared error (MSE). Morris screening based on mean absolute elementary effects revealed output-dependent sensitivities, with cutting thickness dominating the force responses and cutting speed dominating the pulse-number responses within the sampled domain. These results demonstrate the potential of MF-TLMLP for predicting HF numerical cutting responses for the investigated rock properties, parameter ranges, and adjacent double-pick configuration.

## 1. Introduction

Roadheaders and other partial-face tunneling machines are increasingly deployed in civil and mining sectors given their enhanced safety and operational efficiency [1–3]. Conical picks, as essential components of these machines, directly influence equipment reliability and efficiency through their design and arrangement [4–6]. Primary conical pick performance measures include: the mean cutting force (MCF), mean peak cutting force (MPCF), fluctuation index (FI), pulse number (PN), and specific energy (SE) [7,8]. Accurate prediction of these indicators is crucial for designing and manufacturing conical picks, as it significantly impacts the time and budget of excavation projects [9,10].

Currently, the cutting force (CF) of conical picks is measured using linear cutting machines (LCM), which are regarded as effective but costly and not widely available [4,11]. Consequently, significant research attention has focused on CF’s response to variations in rock physical-mechanical properties, pick attributes, and cutting parameters. Various theoretical and empirical prediction models have been developed [12–15]. For instance, a theoretical model based on tensile failure was established by [12] and subsequently refined by [13,14], and [15], incorporating factors such as friction angle and semi-empirical methods to improve accuracy.

The relationship between cutting force and contributing factors has been extensively researched. Both empirical and theoretical frameworks for performance prediction rely heavily on five core parameters: cutting speed *v*, thickness *d*, cone angle ϕ, attack angle γ, and cutting spacing *s* [16–18]. Artificial Neural Network (ANN) techniques were applied by [19] to develop reliable prediction equations for cutting performance assessment. MLPs, a fundamental class of ANNs, are particularly well-suited for this task due to their proven ability as universal approximators, capable of capturing the complex, non-linear relationships between cutting parameters and performance metrics. However, accurate data-driven models require substantial data, which can be categorized by its accuracy and acquisition cost. High-fidelity (HF) data (e.g., from fine-grid simulations or physical experiments) is accurate but costly and time-consuming to acquire [20]. Conversely, low-fidelity (LF) data (e.g., from coarse-grid simulations or simplified models) is cheaper to obtain but less accurate. The scarcity of HF data is a significant bottleneck for training models.

However, LF data only approximate conical pick behavior and are less accurate compared to HF data. Simply combining LF and HF data is insufficient to significantly enhance predictive accuracy [20,21]. To address this limitation, deep transfer learning (TL) methodologies have been implemented, enabling hierarchical neural networks to be trained using multi-fidelity data [22]. This approach proves more promising, as deep TL architectures can be developed upon DNN frameworks. In multilayer DNNs, each layer processes features from preceding layers using activation functions, abstracting information for subsequent layers. Lower-level layers are widely recognized as being less task-specific [23]. This characteristic has motivated TL’s deployment in deep learning to improve efficiency while mitigating overfitting, resulting in networks termed transfer learning neural networks (TLNN). However, standard TLNN approaches often involve simple fine-tuning, which may be insufficient when the correlation between LF and HF data is complex or non-linear. These models need fewer trainable parameters and demonstrate reduced overfitting susceptibility. Nevertheless, if input data are not adequately fused with LF features (rather than just outputs), fine-tuning alone may yield a network with inadequate information for successful convergence [24,25]. This highlights a critical need for more sophisticated fusion mechanisms that explicitly bridge the fidelity gap.

The paper’s subsequent organization is outlined below. Section 2 details the multi-fidelity transfer learning neural networks after a brief overview of common data-driven MLP approaches. Section 3 describes the mechanistic model used to construct the numerical model. Section 4 describes how the simulation model is constructed to generate both LF and HF data. Section 5 compares the performance of MF-TLMLP with other models and predicts the cutting performance of the conical pick. The effect of various parameters on the cutting performance of conical picks is also evaluated. Conclusions are summarised in Section 6.

## 2. MF-TLMLP-based multi-fidelity modeling method for conical picks

### 2.1. Data-driven multi-layer perceptron

A data-driven Multi-Layer Perceptron (MLP) was developed in this study to establish a TL paradigm. The hierarchical structure of MLP was utilized to reconcile the scarcity of HF data with the data-intensive nature of deep learning. Performance indicators for conical picks, including mean cutting force (*MCF*_1_, *MCF*_2_), mean peak cutting force (*MPCF*_1_, *MPCF*_2_), fluctuation index (*FI*_1_, *FI*_2_), pulse number (*PN*_1_, *PN*_2_), and specific energy (*SE*), were defined as MLP output variables. Input parameters, denoted as $X=\left[X_{1},X_{2},...,X_{n}\right]:\Omega \to {\mathbb{R}}^{n}$, encompass cutting speed *v*, thickness *d*, cone angle ϕ, attack angle γ and cutting spacing *s*.

However, MLP-based modeling demands substantial data volumes. As previously noted, this approach is inherently data-hungry. When applied to conical picks with multiple input parameters, obtaining comprehensive experimental data becomes costly and time-intensive due to the need to explore diverse parameter combinations. Consequently, an MLP trained on insufficient data often fails to accurately capture performance under varying conditions. This issue is addressed in the subsequent subsection.

### 2.2. Multi-fidelity transfer learning multi-layer perceptron

TL applies knowledge transfer from a source task to improve target task performance. In MLP trained with ample data, generic features are typically developed in lower layers, while task-specific features emerge in higher layers [23]. To capitalize on this, lower-layer parameters of a pre-trained MLP were frozen, and higher layers were fine-tuned using target-domain data.

This study proposes the Multi-Channel Fusion-Based Multi-Fidelity Transfer Learning Method (MF-TLMLP) to accurately predict conical pick cutting performance by effectively integrating scarce HF data with abundant LF data. The main contributions are: (1) A response-level dual-path formulation integrates the direct MLP prediction with an AE-transformed response for multi-output LF-to-HF transfer. (2) Justification and application of the MLP as a robust baseline for multi-fidelity learning in this domain. (3) A comparative analysis demonstrating that MF-TLMLP achieves superior accuracy and faster convergence than standard MLP and TL approaches.

The methodological contribution examined here is the response-level formulation of multi-fidelity transfer for the nine-output adjacent double-pick problem. Relative to the single-route fine-tuning baseline, MF-TLMLP retains the direct MLP prediction and additionally maps that nine-dimensional response through an AE encoder–decoder. The two response paths are combined within the HF prediction objective. Autoencoder-assisted multi-channel transfer learning has been investigated previously [25]; the present study specifies the response-space coupling, output-layer-only HF adaptation, and simultaneous prediction of nine cutting responses within the investigated coarse- and fine-mesh numerical setting. The contribution is therefore defined by this coordinated formulation and its evaluation for the stated prediction problem. The resulting multi-fidelity framework is termed MF-TLMLP. Its architecture is illustrated in Fig 1, and the implementation is described below.

**Fig 1:**
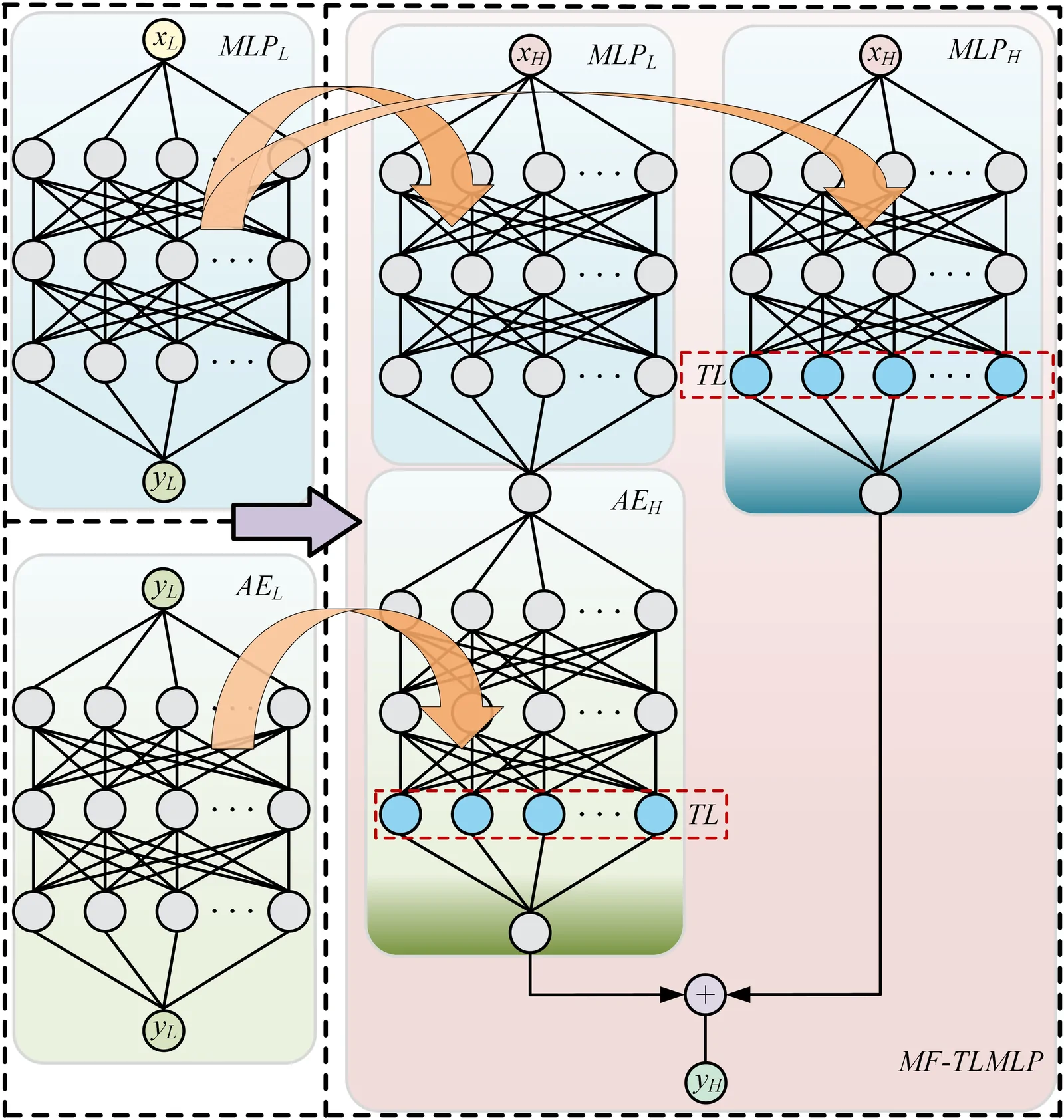
The left section depicts the LF training stage with $MLP_{L}$ (top) and $AE_{L}$ (bottom). The right section illustrates the MF-TLMLP training stage, integrating multi-fidelity data fusion and TL via a multi-channel approach. Most parameters are fixed during HF training, with only the final layers.

The pre-trained MLP and AE were used to initialize the HF prediction model. For an HF input, the five physical variables are expanded into 20 polynomial features and min–max normalized, giving the preprocessed input vector ${\tilde{x}}_{H}\in {\mathbb{R}}^{20}$.

The direct MLP pathway, denoted by *Q*, produces a nine-dimensional response vector. This response vector is also supplied to the AE pathway, denoted by *P*, which comprises an encoder and a decoder and maps a nine-dimensional input to a nine-dimensional output.

At inference, with dropout disabled, the prediction is expressed as


$$\begin{array}{cc} u & =Q({\tilde{x}}_{H}),\\ {\hat{\tilde{y}}}_{H} & =(1-\beta )u+\beta P(u),\;\beta =0.5. \end{array}$$ (1)

Here, $Q:{\mathbb{R}}^{20}\to {\mathbb{R}}^{9}$ denotes the complete MLP mapping, and $P:{\mathbb{R}}^{9}\to {\mathbb{R}}^{9}$ denotes the AE encoder–decoder mapping. Both pathways produce predictions for the same nine response components: *MCF*_1_, *MCF*_2_, *MPCF*_1_, *MPCF*_2_, *FI*_1_, *FI*_2_, *PN*_1_, *PN*_2_, and *SE*. Fusion is performed component by component in the normalized response space, and the final predictions are transformed back to their physical scales using the inverse target normalization.

During training, the direct and AE pathways use separate forward evaluations of the same MLP, sharing its parameters but using separate dropout masks.

The fusion coefficient was fixed at β=0.5 within the jointly fine-tuned model, rather than estimated from the individual predictive accuracy of the two pathways. The MLP output layer and the final AE decoder layer were jointly updated using the MSE of the fused prediction over the nine individually min–max-normalized HF outputs. Although the mixing coefficients were fixed, the terminal layers retained output-specific weights and biases, allowing the response mappings to adapt across output variables. This symmetric averaging rule avoids introducing additional mixing parameters. Output-specific or learnable fusion coefficients represent a potential extension of the present framework.

## 3. Mechanistic model

The Drucker-Prager intrinsic model, implemented in LS-DYNA, was selected for this study. Unlike the Mohr-Coulomb model, the Drucker-Prager model avoids singularities in the yield surface and provides a smoother numerical implementation, which enhances convergence in dynamic finite element simulations. It is a pressure-dependent plasticity model widely employed for simulating mechanical responses in geomaterials, including soils, rocks, and concrete. This model extends the classical von Mises yield criterion by incorporating hydrostatic stress sensitivity, making it particularly suitable for modeling the nonlinear deformation and failure of coal-rock under complex stress states during pick cutting. The adopted failure criterion represents a three-dimensional, pressure-dependent formulation that forecasts rock stress states at ultimate strength [26]. Its mathematical expression is:


$$\begin{array}{c} \sqrt{J_{2}}=\lambda {I^{\prime }}_{1}+\kappa \end{array}$$ (2)

where λ and κ are material constants, *J*_2_ is the second invariant of the stress deviator tensor, and $I_{1}^{\prime }$ is the first invariant of the stress tensor, defined as:


$$\begin{array}{c} {I^{\prime }}_{1}={\sigma^{\prime }}_{1}+{\sigma^{\prime }}_{2}+{\sigma^{\prime }}_{3} \end{array}$$ (3)

$$\begin{array}{c} J_{2}=\frac{1}{6}\left[(\sigma_{1}^{\prime }-\sigma_{2}^{\prime }{)}^{2}+(\sigma_{2}^{\prime }-\sigma_{3}^{\prime }{)}^{2}+(\sigma_{3}^{\prime }-\sigma_{1}^{\prime }{)}^{2}\right]. \end{array}$$ (4)

where $\sigma_{1}^{\prime }$, $\sigma_{2}^{\prime }$, and $\sigma_{3}^{\prime }$ represent principal effective stresses.

This formulation captures the pressure-dependent plasticity inherent in coal rocks, making it an apt choice for simulating their response to the dynamic loading imposed by conical picks. Essential to the Drucker-Prager model, parameters λ and κ are calculated from the rock’s cohesion *c* and internal friction angle φ. These values are established via triaxial tests or derived from standard compression tests using:


$$\begin{array}{c} \lambda =\frac{2\sin\varphi }{\sqrt{3}\left(3-\sin\varphi \right)} \end{array}$$ (5)

$$\begin{array}{c} \kappa =\frac{6c\cos\varphi }{\sqrt{3}\left(3-\sin\varphi \right)} \end{array}$$ (6)

where *c* and φ denote the cohesion intercept and internal friction angle of the rock, respectively.

This constitutive model was implemented within LS-DYNA to replicate the rock’s mechanical response during the cutting process. The choice of the Drucker-Prager intrinsic model within LS-DYNA facilitated the simulation of complex stress states induced by the stress-coupled response at the conical pick/coal rock interface, capturing both elastic deformation and plastic yielding. Parameters such as cohesion *c* and friction angle φ were input directly into the material model, derived from the experimental strengths, while the software’s built-in algorithms handled the numerical integration of the plasticity equations. During the simulation, the stress state at each time step was evaluated against the yield criterion. If this was exceeded, plastic strains were computed to adjust the stress back to the yield surface. This ensured a realistic representation of the rock’s failure under cutting loads. This approach provided a robust theoretical foundation for analyzing the pick-cutting process, linking the physical properties of the coal rock to its performance under operational conditions.

## 4. Finite element model construction

To investigate the effects of cutting speed *v*, thickness *d*, cone angle ϕ, attack angle γ and cutting spacing *s* on conical pick performance, a numerical simulation of adjacent double-pick cutting was established. Simulations were conducted across ranges of cutting speed (1~3 m/s), thickness (5~30 mm), cone angles ($60^{\circ }$, $80^{\circ }$, $100^{\circ }$), attack angles ($40^{\circ }\sim 60^{\circ }$), and cutting spacing (5~50 mm). The geometric configurations and mesh discretizations of the LF and HF finite element models for adjacent double-pick cutting are illustrated in Fig 2.

**Fig 2:**
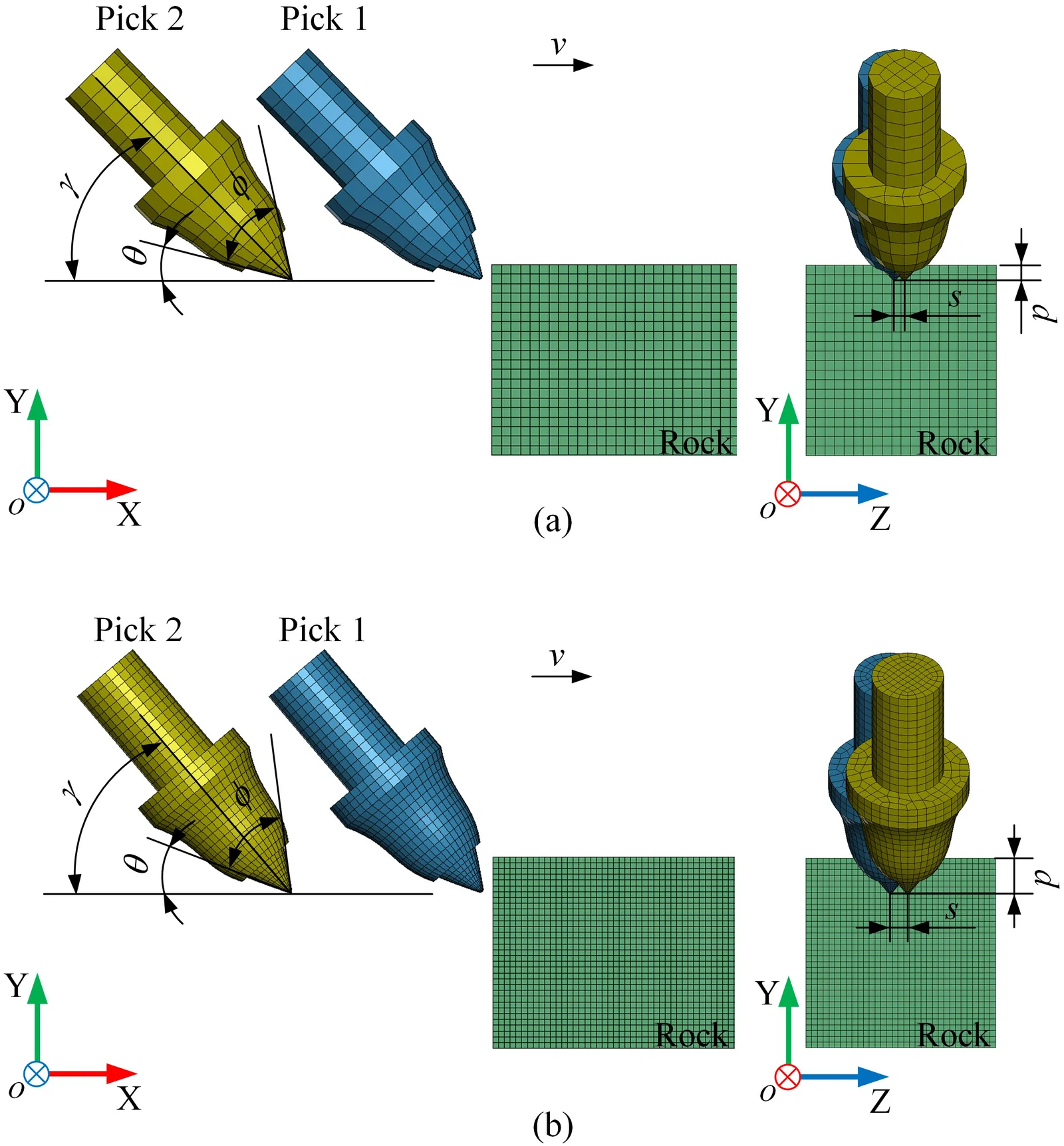
Finite element model of adjacent double-pick cutting. (a) LF model with 60° cone angle; (b) HF model with 60° cone angle.

Latin Hypercube Sampling (LHS) was utilized to ensure comprehensive coverage of the design space, providing a more uniform and representative sample distribution than random sampling [27–29]. Input parameters were sampled using LHS for 2500 LF and 500 HF models. LF samples were generated using 2500 parameter sets, while HF samples used 500 sets. The number of samples for each fidelity level was determined by a trade-off between design space coverage and computational cost. As detailed later in this section, a single HF simulation required approximately 200 minutes, whereas an LF simulation completed in 7 minutes. Generating a large HF dataset was thus computationally prohibitive, so 500 HF samples were chosen as a feasible dataset for fine-tuning, while 2500 LF samples provided a robust dataset for pre-training. For LF models, picks were meshed rotationally to maintain consistent cone angles, with 540 cells and 788 nodes for $60^{\circ }$ and $80^{\circ }$ picks, and 416 cells and 694 nodes for $100^{\circ }$ picks. The pick material was defined as rigid to accurately simulate contact with the rock while optimizing computational efficiency. The rock model, measuring 500mm×100mm×100mm, was meshed with hexahedral elements at 5 mm spacing, yielding 40,000 elements and 44,541 nodes. This ensured sufficient computational accuracy at a minimum cutting depth of 5 mm. Displacement constraints were applied in the X, Y, and Z directions to the rock’s lower surface and in the X direction to its rear surface. Non-reflective boundary conditions were added to the sides and lower surfaces to minimize model size effects.

For HF models, finer meshes were employed: 4316 cells and 5285 nodes for $60^{\circ }$ picks, 4318 cells and 5294 nodes for $80^{\circ }$ picks, and 4196 cells and 5135 nodes for $100^{\circ }$ picks. The rock model was meshed at 3 mm spacing, resulting in 181863 elements and 194,208 nodes. Simulations were performed on a workstation with an AMD Ryzen 7950X @ 4.5 GHz CPU and 64 GB RAM, with computation times of approximately 200 minutes for HF models and 7 minutes for LF models.

The rock’s mechanical properties are shown in Table 1 [30]. Stress distributions are depicted in Fig 3. The LF and HF von Mises equivalent-stress contours are displayed using a common color scale for comparison. However, the HF model (b) clearly resolves a more localized stress concentration at the pick tip and a more defined fracture propagation zone, whereas the LF model (a) shows a more diffuse, widespread stress-affected area. This qualitative difference in stress resolution is the primary reason for the discrepancy in cutting force predictions.

**Table 1:** Mechanical properties of the rock.

Parameter	Value
Density (ρ, g/cm³)	2.22
Uniaxial compressive strength ($\sigma_{c}$, MPa)	17.91
Tensile strength ($\sigma_{t}$, MPa)	1.64
Tangent Young’s modulus (*E*, GPa)	3.00

**Fig 3:**
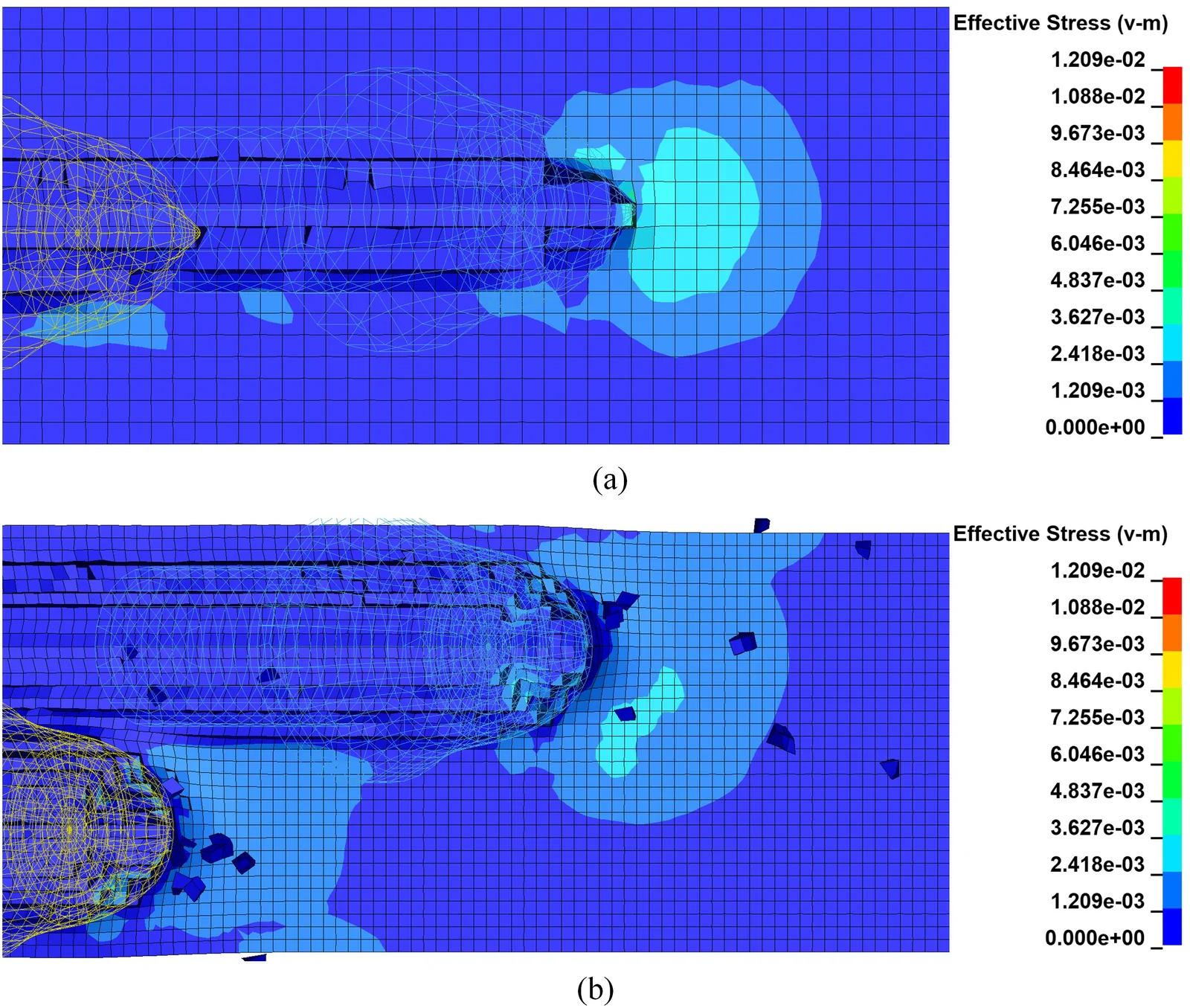
Stresses for models with different fidelity. (a) LF model, (b) HF model.

The simulation results for the double-pick cutting forces under different model fidelity levels are shown in Fig 4. Pick1 was observed to exhibit 12%−15% higher cutting forces than Pick2 across both fidelity models. This difference is attributed to their cutting order, as seen in Fig 4. Therefore, Pick 2 interacts with rock that has already been stressed and potentially pre-fractured by the passage of Pick 1, resulting in a lower required cutting force. Notably, the lower cutting depth in Model 1 (Fig 4(a)) contributed to producing more stable force oscillations compared to Model 2.

**Fig 4:**
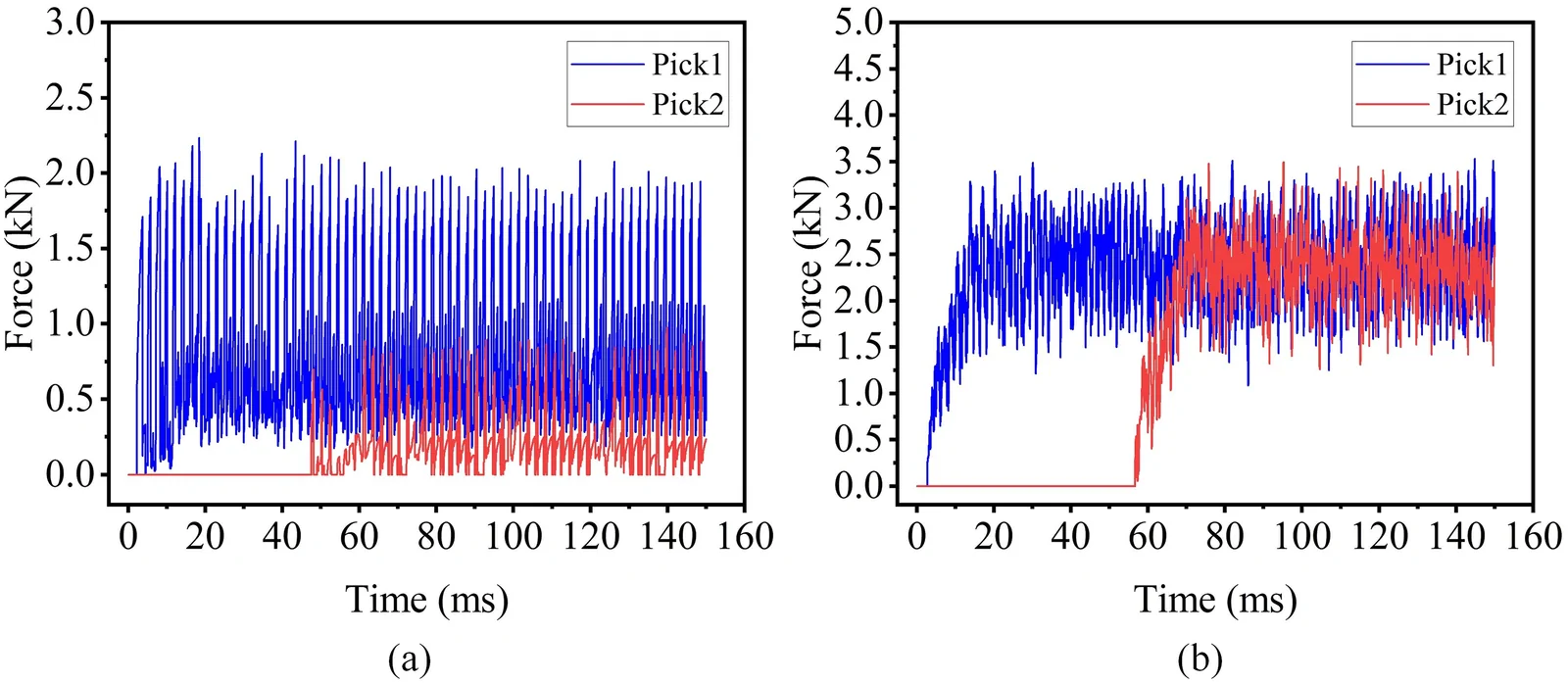
Cutting forces for different models. (a) LF model, (b) HF model.

During simulations, cutting forces and rock fracture characteristics were recorded. Subsequently, MCF, MPCF, FI, PN, and SE were calculated. MCF represents the average cutting force, while MPCF is the average of peaks over 50 data points. FI is a dimensionless parameter for evaluating cutting force fluctuation intensity [7,31].


$$\begin{array}{c} FI_{1}=\frac{1}{\overset{―}{F}c_{1}}\cdot \frac{1}{n}\sum_{i=1}^{n}\left(Fc_{i1}-\overset{―}{F}c_{1}\right) \end{array}$$ (7)

$$\begin{array}{c} FI_{2}=\frac{1}{\overset{―}{F}c_{2}}\cdot \frac{1}{n}\sum_{i=1}^{n}\left(Fc_{i2}-\overset{―}{F}c_{2}\right) \end{array}$$ (8)

where ${\overset{―}{F}}_{c1}$ and ${\overset{―}{F}}_{c2}$ are the Mean Cutting Force (MCF) for Pick 1 and Pick 2, respectively, and *n* is the total number of data points. PN quantifies peak frequency per unit displacement and is employed to assess cutting force fluctuation frequency, defined by Eq. 9 and Eq. 10.


$$\begin{array}{c} PN_{1}=\frac{m_{1}}{l_{1}} \end{array}$$ (9)

$$\begin{array}{c} PN_{2}=\frac{m_{2}}{l_{2}} \end{array}$$ (10)

where *m*_1_ is the total number of peak forces for Pick 1 exceeding a threshold $\overset{―}{F}c_{1}/2$, *l*_1_ is the cutting displacement of the Pick 1, *m*_2_ is the tota*l* number of peak forces for Pick 2 exceeding a threshold $\overset{―}{F}c_{2}/2$, and *l*_2_ is the cutting displacement of Pick 2.

High FI and PN values indicate severe load fluctuations, which can accelerate tool fatigue. SE (the energy consumed per unit volume of fractured rock) is expressed by:


$$\begin{array}{c} SE=\frac{W}{V_{c}}=\frac{\int_{0}^{l}Fc\;dx}{V_{c}} \end{array}$$ (11)

where *W* is the work by integrating cutting force over displacement, and $V_{c}$ is the chip volume.

Because the LF and HF sampling designs were non-nested, cross-fidelity associations were assessed using input-proximity matching. Cone angle was matched exactly, while the normalized difference in each remaining input was limited to 5% of its design range. An output-blind, one-to-one assignment maximized the number of admissible pairs and minimized their total normalized Euclidean distance. This yielded 33 pairs. Pearson and Spearman coefficients were calculated for all nine responses, and the analysis was repeated using 7.5% and 10% calipers, yielding 161 and 321 pairs, respectively. The results are summarized in Table 2.

**Table 2:** LF–HF correlations from input-proximity matching.

Response	*r*, 5%	ρ, 5%	*r*, 7.5%	*r*, 10%
*MCF*_1_	0.952	0.916	0.966	0.958
*MCF*_2_	0.932	0.928	0.946	0.934
*MPCF*_1_	0.955	0.929	0.962	0.947
*MPCF*_2_	0.946	0.918	0.940	0.924
*FI*_1_	0.610	0.418	0.548	0.546
*FI*_2_	0.337	0.364	0.524	0.472
*PN*_1_	0.603	0.543	0.743	0.738
*PN*_2_	0.667	0.647	0.681	0.675
*SE*	0.656	0.595	0.810	0.713

*r*: Pearson correlation coefficient; ρ: Spearman rank correlation coefficient. The 5%, 7.5%, and 10% calipers produced 33, 161, and 321 pairs, respectively. Cone angle was matched exactly; each other input difference was bounded by the stated percentage of its design range. Each source sample was used at most once within each matched set. The matched inputs were close but not identical.

For the 5% caliper, the force-related responses exhibited Pearson coefficients of 0.932–0.955 and Spearman coefficients of 0.916–0.929. Weaker associations were observed for the remaining responses. The results indicate shared cross-fidelity response trends, particularly for cutting forces.

## 5. Construction and training of the multi-fidelity neural model

### 5.1. Pre-training of MLP and AE for the LF model

A MLP was pre-trained using LF data obtained from 2500 LF model simulations. The input vectors were defined as $X_{L}=\{v,d,\phi ,\gamma ,s\}$, representing cutting speed, thickness, cone angle, attack angle, and cutting spacing, respectively. The output vectors were specified as $Y_{L}=\{MCF_{1},MCF_{2},MPCF_{1},MPCF_{2},FI_{1},FI_{2},PN_{1},PN_{2},SE\}$, encompassing mean cutting force, mean peak cutting force, fluctuation index, pulse number, and specific energy for dual-pick. The MLP architecture is illustrated in Fig 5.

**Fig 5:**
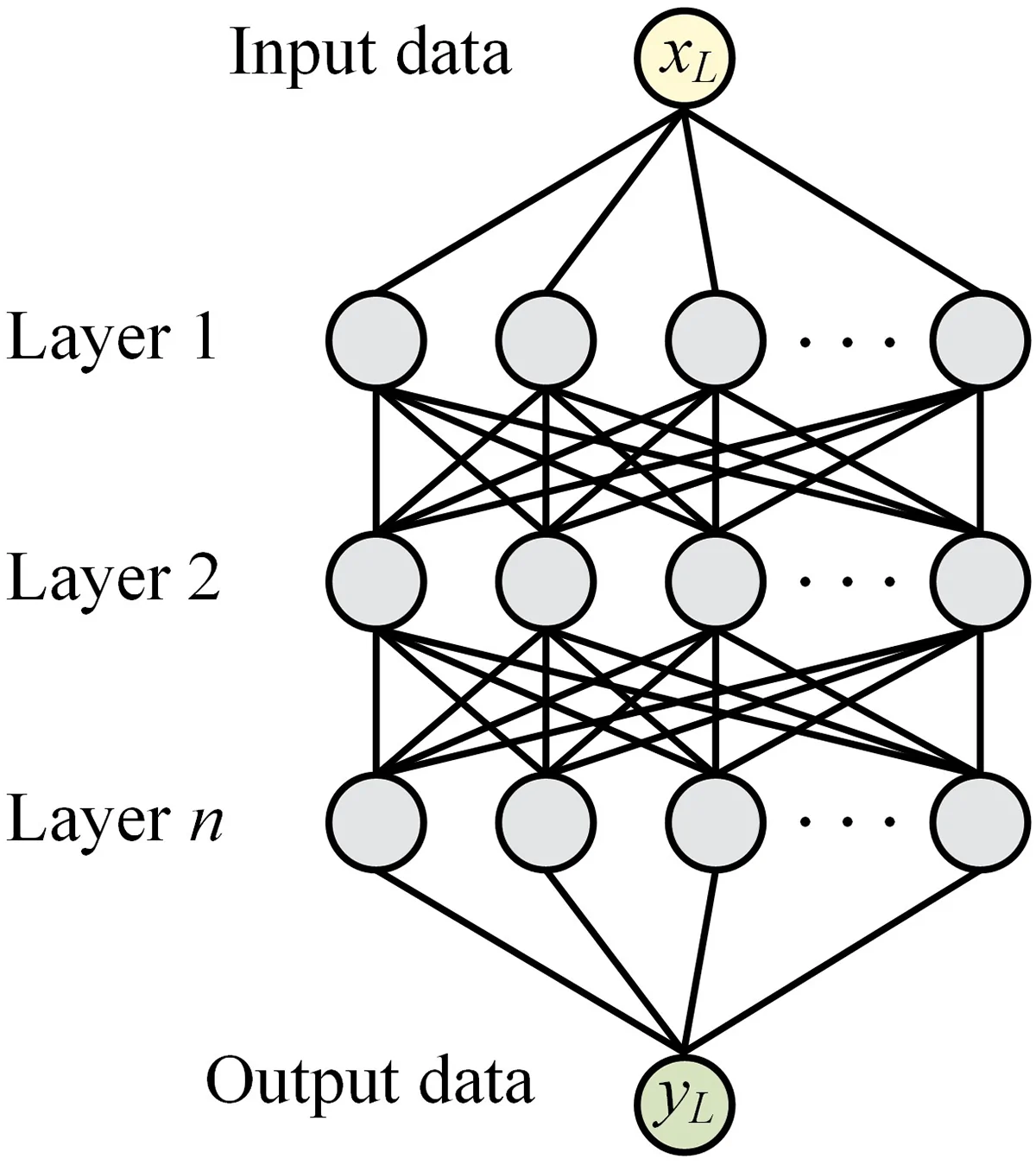
Architecture of MLP model.

Hyperparameter optimization was performed using Bayesian Optimization (BO) [32,33] to minimize validation error. A systematic approach was adopted to adjust the key hyperparameters, which included the number of neurons, the number of layers, and the learning rate. The Adam optimization algorithm was employed for model training, with 2000 samples (80% of the 2500 LF samples) assigned to training and validation, while the remaining 500 samples (20%) were allocated for testing. To ensure robustness, *K*-fold cross-validation (*K* = 5) was applied [34]. The five physical input variables were expanded into 20 second-order polynomial features without a constant term, comprising five original variables, five squared terms, and ten pairwise products. Min–max scaling was then applied to the expanded input features [35]. The optimized hyperparameters are presented in Table 3.

**Table 3:** Pre-tuning hyperparameter results of the MLP model.

Hyperparameters	Lower bound	Tuning result	Upper bound
Number of neurons	50	186	1000
Number of layers	1	2	5
Learning rate	1e-6	4.014e-4	0.01
Dropout rate	0.0	0.028	0.5
Mini-batch size	32	128	128
*L*_2_ regularization factor	1e-6	1.007e-6	0.01

The layer-wise architecture used for HF adaptation is summarized in Table 4.

**Table 4:** Layer-wise architecture of the MF-TLMLP model used for HF adaptation.

Module	Dimensions	Operations
MLP hidden layer 1	20→186	Fully connected, BatchNorm, ReLU, Dropout
MLP hidden layer 2	186→186	Fully connected, BatchNorm, ReLU, Dropout
MLP output layer	186→9	Fully connected, ReLU
AE encoder	9→186→186→9	ReLU after both 186-dimensional layers; linear final layer
AE decoder	9→186→186→9	ReLU after both 186-dimensional layers; linear final layer
Output fusion	9+9→9	Element-wise weighted average with β=0.5

The MLP dropout rate is 0.02820579. During HF adaptation, only the MLP output-layer parameters and the final AE decoder-layer parameters are updated by gradient-based optimization. Batch-normalization running statistics continue to update during training.

The predictive accuracy for the MLP was evaluated using mean squared error (MSE) and the coefficient of determination (R^2^) on the test set. These metrics, grounded in standard equations, quantify the model’s performance by comparing actual and predicted values of the output variables. Test set prediction errors were determined based on MSE and R^2^ values to validate the accuracy of the constructed MLP, as defined by Eq. 12 and Eq. 13.


$$\begin{array}{c} MSE=\frac{1}{n}\sum_{i}^{N}{\left(y_{actual}^{i}-y_{pred}^{i}\right)}^{2} \end{array}$$ (12)

$$\begin{array}{c} R^{2}=1-\frac{\sum_{i}^{N}{\left(y_{actual}^{i}-y_{pred}^{i}\right)}^{2}}{\sum_{i}^{N}{\left(y_{actual}^{i}-\overset{―}{y_{actual}^{i}}\right)}^{2}} \end{array}$$ (13)

where $y_{actual}^{i}$, $y_{pred}^{i}$ and $\overset{―}{y_{actual}^{i}}$ are the actual, predicted and average values of the respective output variables *MCF*_1_, *MCF*_2_.

The simulations were executed on a workstation equipped with an Intel 13700K CPU at 3.4 GHz, 64 GB RAM, and an Nvidia 4090 GPU with 24 GB memory. The BO process required 1207 seconds, while the MLP training was completed in 0.66 seconds. The AE comprises an encoder and a decoder with nine-dimensional input and output interfaces, as detailed in Table 4. During HF adaptation, the AE receives the MLP response vector and produces a response transformation that contributes to the final fused prediction.

### 5.2. Construction of the multi-fidelity transfer model using deep TL

The MLP pre-trained on extensive low-fidelity data (source domain) was fine-tuned using high-fidelity data (target domain) for constructing a multi-fidelity surrogate model. This adapted model retained the original MLP’s architectural framework.

During HF fine-tuning, only the weights and biases of the MLP output layer and the final fully connected layer of the AE decoder were jointly updated. Both MLP hidden layers, the AE encoder, and the preceding AE decoder layers were excluded from gradient-based parameter updates. Batch-normalization running means and variances continued to update during training.

Adam was used with a learning rate of $4.013783\times 10^{-4}$ and a weight-decay coefficient of $1.010817\times 10^{-8}$. The objective was the MSE of the fused prediction over the nine individually min–max-normalized HF responses. No separate AE reconstruction loss was used during HF fine-tuning.

As depicted in Fig 6, the MF-TLMLP algorithm demonstrated superior performance compared to traditional MLP and TL algorithms in terms of MSE. Specifically, as shown in Fig 6(a) and 6(b), the MF-TLMLP consistently outperformed MLP and TL algorithms in terms of MSE when using 500 samples. For instance, in the case of 500 samples, MF-TLMLP achieved significantly lower MSE values across the epochs, whereas the MLP500 algorithm resulted in noticeably higher MSE values. This suggests that MF-TLMLP could reduce prediction errors more effectively, especially in larger sample sizes. Furthermore, MF-TLMLP showed faster convergence, as evidenced by the rapid decrease in MSE over epochs. This indicates that the algorithm learned faster and captured underlying data patterns more efficiently than the other models. The faster decline in MSE curves in the inset of Fig 6(b) highlights this superior performance.

**Fig 6:**
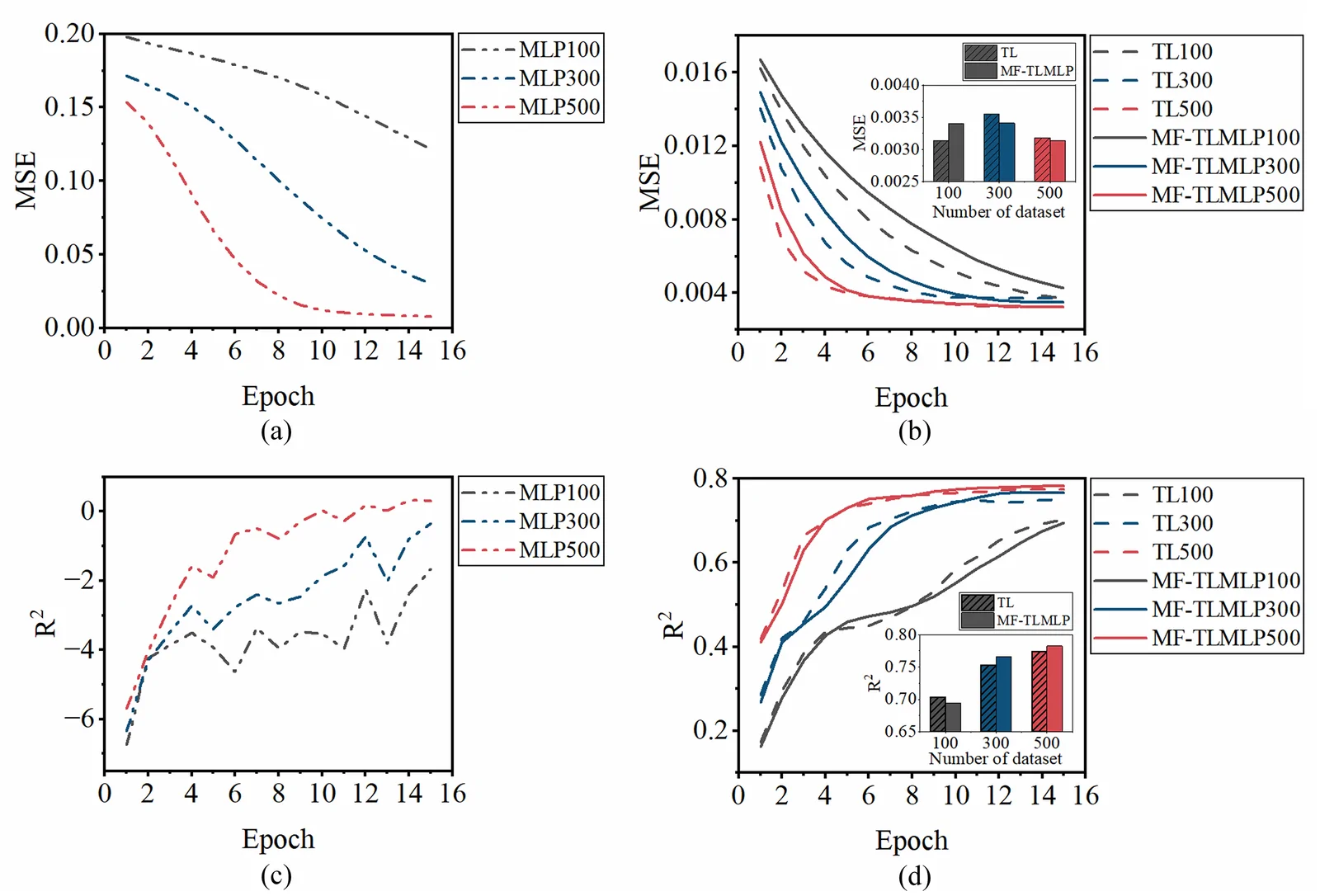
Comparison of the training process of MLP, TL and MF-TLMLP models in terms of: (a) MSE for MLP; (b) MSE for TL and MF-TLMLP; (c) R^2^ for MLP; (d) R^2^ for TL and MF-TLMLP.

Nonetheless, MF-TLMLP did not consistently outperform the MLP and TL algorithms in all scenarios. For example, with 100 samples as shown in Fig 6(a) and 6(d), MF-TLMLP did not significantly outperform TL100 or MLP100 in terms of both MSE and R^2^. In this case, the advantages of MF-TLMLP are less obvious, suggesting that the performance benefits of the complex fusion architecture may not be realized when the HF data is extremely scarce.

This comparison highlights a critical finding: while all models struggle when HF data is extremely scarce (n = 100), the advantages of the MF-TLMLP architecture become highly significant as the dataset size reaches a more practical, albeit still limited, level (n = 500). This demonstrates that MF-TLMLP is particularly effective at leveraging modestly scarce HF datasets, maximizing information gain where traditional MLP and simpler TL models fail to generalize. The robust performance at n = 500 validates the model’s efficiency for the typical engineering scenario where HF data is costly and limited, but not non-existent, as depicted in the more robust performance metrics shown in Fig 6(c) and Fig 6(d).

### 5.3. Influence of cutting parameters on cutting performance

Sensitivity analysis was conducted to assess the effects of input variations on the cutting-performance responses predicted by MF-TLMLP within the investigated numerical domain. This approach enabled the identification of influential factors shaping conical pick performance. Using the MF-TLMLP model, the impact of cutting speed *v*, thickness *d*, cone angle ϕ, attack angle γ, and cutting spacing *s* on nine performance metrics (*MCF*_1_, *MCF*_2_, *MPCF*_1_, *MPCF*_2_, *FI*_1_, *FI*_2_, *PN*_1_, *PN*_2_, *SE*) was examined.

The Morris elementary-effects approach was used to screen the five input parameters [36,37]. The analysis retained the first 12 trajectories in the archived design, corresponding to 72 evaluated input vectors and 12 elementary effects per parameter. Each trajectory perturbed each parameter once on an eight-level grid. The inputs were mapped to the unit hypercube using $z_{i}=(x_{i}-a_{i})/(b_{i}-a_{i})$, where $a_{i}$ and $b_{i}$ are the bounds given in Table 5. The archived trajectories used adjacent grid steps of magnitude 1/(8−1)=1/7 in normalized coordinates. The actual signed increment was retained when calculating each elementary effect.

**Table 5:** Input ranges used in the surrogate-based Morris sensitivity analysis.

Parameter	Unit	Lower bound	Upper bound
Cutting speed, *v*	m/s	1	3
Cutting thickness, *d*	mm	5	30
Cone angle, ϕ	°	60	100
Attack angle, γ	°	40	60
Cutting spacing, *s*	mm	5	50

For response *j* and input *i*, the elementary effect along trajectory *t* was calculated as


$$\begin{array}{c} EE_{ij}^{\left(t\right)}=\frac{{\hat{f}}_{j}(z_{i}^{\left(t,1\right)})-{\hat{f}}_{j}(z_{i}^{\left(t,0\right)})}{z_{i}^{\left(t,1\right)}-z_{i}^{\left(t,0\right)}}, \end{array}$$ (14)

where $z_{i}^{\left(t,0\right)}$ and $z_{i}^{\left(t,1\right)}$ denote successive trajectory points differing only in input *i*, and ${\hat{f}}_{j}$ denotes the surrogate response expressed as a function of the normalized inputs.

Three statistics were calculated:


$$\begin{array}{cc} \mu_{ij} & =\frac{1}{r}\sum_{t=1}^{r}EE_{ij}^{\left(t\right)},\\ \mu_{ij}^{*} & =\frac{1}{r}\sum_{t=1}^{r}\left|EE_{ij}^{\left(t\right)}\right|,\\ \sigma_{ij} & ={\left[\frac{1}{r-1}\sum_{t=1}^{r}{\left(EE_{ij}^{\left(t\right)}-\mu_{ij}\right)}^{2}\right]}^{1/2}, \end{array}$$ (15)

with *r* = 12.

The mean absolute effect $\mu^{*}$ was used to rank overall parameter influence without cancellation between positive and negative effects. The signed mean μ was retained to describe the average direction, while σ described variation across sampled conditions consistent with nonlinearity and/or interactions; it does not isolate individual interaction effects.

For presentation, $\mu_{ij}^{*}$ and $\sigma_{ij}$ were divided by the same quantity, $\max_{k}\mu_{kj}^{*}$, separately for each response. The relative $\mu^{*}$ values are shown in Fig 7. Both statistics for all five inputs and nine responses are reported in Table 6. Each entry lists the relative $\mu^{*}$ followed by the correspondingly scaled σ in parentheses. These values describe within-response relative effects.

**Fig 7:**
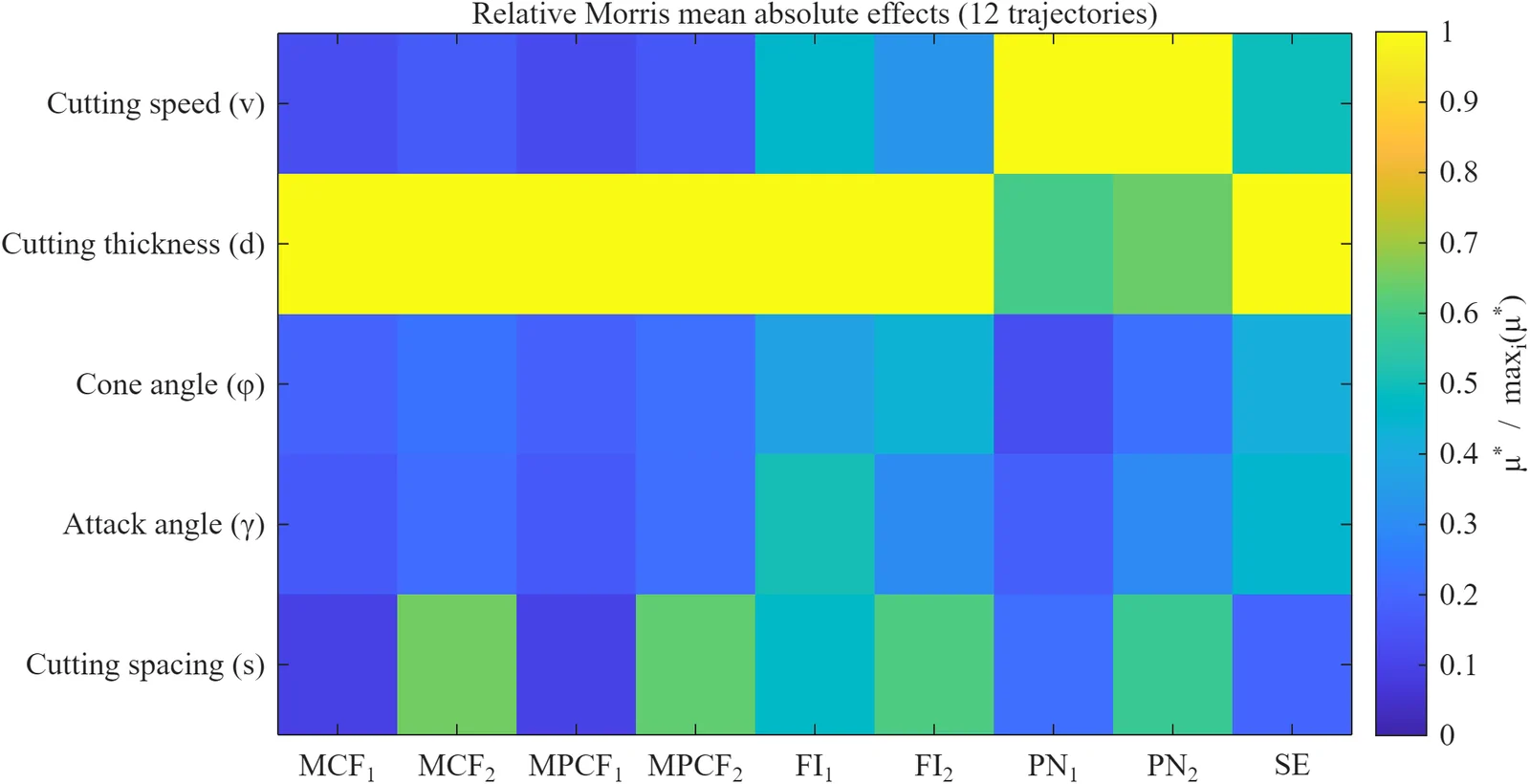
Relative mean absolute elementary effects for the five input parameters and nine cutting-performance responses, based on 12 trajectories. Each response column is divided by its own maximum $\mu^{*}$ across the five inputs.

**Table 6:** Relative mean absolute elementary effects and standard deviations based on 12 trajectories.

Response	*v*	*d*	ϕ	γ	*s*
*MCF*_1_	0.131 (0.153)	1.000 (0.166)	0.189 (0.127)	0.166 (0.135)	0.096 (0.130)
*MCF*_2_	0.169 (0.187)	1.000 (0.322)	0.233 (0.176)	0.218 (0.166)	0.649 (0.455)
*MPCF*_1_	0.122 (0.102)	1.000 (0.176)	0.186 (0.128)	0.165 (0.119)	0.099 (0.138)
*MPCF*_2_	0.160 (0.155)	1.000 (0.282)	0.223 (0.156)	0.220 (0.172)	0.630 (0.435)
*FI*_1_	0.460 (0.609)	1.000 (1.056)	0.370 (0.389)	0.504 (0.625)	0.471 (0.561)
*FI*_2_	0.332 (0.500)	1.000 (1.062)	0.435 (0.419)	0.300 (0.266)	0.609 (0.866)
*PN*_1_	1.000 (0.636)	0.596 (0.358)	0.134 (0.164)	0.181 (0.297)	0.221 (0.318)
*PN*_2_	1.000 (0.505)	0.642 (0.627)	0.227 (0.340)	0.295 (0.345)	0.578 (0.674)
*SE*	0.496 (0.414)	1.000 (1.024)	0.418 (0.395)	0.451 (0.438)	0.199 (0.256)

Each entry gives $\mu_{ij}^{*}/m_{j}$ followed by $\sigma_{ij}/m_{j}$ in parentheses, where $m_{j}=\max_{k}\mu_{kj}^{*}$ over the five inputs. The same positive scale is used for both statistics within each response. Parenthesized values are scaled standard deviations, not standard errors or confidence intervals; they can exceed one. Values do not represent fractions of output variance. *v*: cutting speed; *d*: cutting thickness; ϕ: cone angle; γ: attack angle; *s*: cutting spacing.

The revised screening results show output-dependent parameter importance. Cutting thickness had the largest $\mu^{*}$ for the four force responses, both fluctuation indices, and specific energy, whereas cutting speed had the largest $\mu^{*}$ for both pulse-number responses. Cutting spacing ranked second for *MCF*_2_, *MPCF*_2_, and *FI*_2_.

For the cone-angle effect on *PN*_2_, the signed mean was μ=44.343, while $\mu^{*}=480.618$ and σ=720.493, illustrating cancellation in the signed mean and appreciable variation among the sampled elementary effects.

These measures characterize the sensitivity of the fitted surrogate within the specified input domain rather than experimentally established interactions.

## 6. Conclusion

This study introduces an MF-TLMLP framework for characterizing dual-pick cutting performance. The methodology employs LS-DYNA simulations, where low-fidelity data originate from coarse-grid simulations while high-fidelity data derive from fine-grid simulations based on deep transfer learning principles. The procedure comprises dual stages. First, dual low-fidelity models are constructed: an $MLP_{L}$ and an $AE_{L}$. Both models exhibit analogous architectures. Subsequently, a dual-channel transfer learning framework is developed. These low-fidelity models are fine-tuned and integrated, enabling simultaneous processing of multi-fidelity data for optimal information utilization. The developed dual-pick cutting model implements this multi-fidelity approach, combining LF model with HF model to define nonlinear relationships between input parameters (cutting speed, thickness, cone angle, attack angle, pick spacing) and performance metrics (MCF, MPCF, FI, PN, SE). Within the investigated numerical domain, the predictive performance of MF-TLMLP was evaluated against a single-fidelity MLP and a conventional transfer-learning model using the HF finite element results as the common reference. Key findings include:

MF-TLMLP reduced prediction errors more effectively, especially in larger sample sizes. Furthermore, MF-TLMLP showed faster convergence, indicating that the algorithm learned faster and captured underlying data patterns more efficiently than MLP and TL. The results support the use of MF-TLMLP as a surrogate for HF numerical predictions of adjacent double-pick cutting within the investigated parameter ranges and for the rock properties considered.

The present conclusions are limited to the numerical simulations for the rock properties, cutting-parameter ranges, and adjacent double-pick configuration considered in this study. Extension to other rock types, complex multi-pick systems, or broader operating conditions remains a direction for future work and requires additional validation. A dedicated conical-pick cutting test programme is currently under preparation to assess the predictions under matched experimental conditions.

## Supporting information

S1 Data The archive contains the LF and HF datasets in Dataset, representative cutting-force records from one LF simulation and one HF simulation in Force.xlsx, loss histories recorded during model training in Loss.xlsx, the corresponding coefficient-of-determination histories in R2.xlsx, and the tabulated results of the revised Morris sensitivity analysis in Main_r12_normalized.csv. (ZIP)
